# The Association between Social Support and Pre-Exposure Prophylaxis use among Sexual Minority Men in the United States: A Scoping Literature Review

**DOI:** 10.1007/s10461-024-04446-4

**Published:** 2024-07-23

**Authors:** Carrie L. Nacht, Hannah E. Reynolds, Owen Jessup, Marianna Amato, Erik D. Storholm

**Affiliations:** 1 School of Public Health, San Diego State University, San Diego, CA USA; 2 Herbert Wertheim School of Public Health and Human Longevity Science, University of California, La Jolla, San Diego, CA USA; 3 College of Education, San Diego State University, San Diego, CA USA

**Keywords:** HIV, Pre-exposure prophylaxis, Sexual minority men, Social Support

## Abstract

Sexual minority men (SMM) are disproportionately affected by HIV. Although pre-exposure prophylaxis (PrEP) is an effective way of reducing HIV incidence, PrEP use has remained relatively low. Social support may be one effective factor in increasing PrEP use among SMM, but the association between social support and PrEP use/adherence is not well understood. The objective of this paper was to summarize the current literature on the association of social support and PrEP use among SMM in the United States. A systematic search was conducted using six different databases MEDLINE / PubMed, PsycINFO, Cochrane CENTRAL, Google Scholar, Embase, and Web of Science using terms established from keywords and medical subject headings (MeSH) terms before being adapted to each database. Data were extracted for key study factors (e.g., study population, geographic location, study design) and main findings. This search produced eleven articles: ten manuscripts and one conference abstract. Of these, two were randomized control trials, two were interventions, three were qualitative, and four were cross-sectional. The studies were widespread across the country, but most were in major metropolitan areas. From the articles included in this review, findings were inconsistent in the association between social support; some studies showed null findings, others that only certain sources of social support were significant, and others that there was a significant association between social support and PrEP use. This review highlights the complexity of the relationship between social support and PrEP use among SMM, indicating the need for further research to identify specific types and sources of support that effectively enhance PrEP uptake and adherence. Targeted interventions based on these insights could significantly reduce HIV incidence in the population.

## Resumen

Los hombres pertenecientes a minorías sexuales (HSH) se ven afectados de forma desproporcionada por el VIH. Aunque la profilaxis preexposición (PrEP) es una forma eficaz de reducir la incidencia del VIH, el uso de la PrEP se ha mantenido relativamente bajo. El apoyo social puede ser un factor eficaz para aumentar el uso de la PrEP entre los SMM, pero la asociación entre el apoyo social y el uso/adherencia a la PrEP no se conoce bien. El objetivo de este documento fue resumir la literatura actual sobre la asociación entre el apoyo social y el uso de la PrEP entre los HSH en los Estados Unidos. Se realizó una búsqueda sistemática en seis bases de datos diferentes MEDLINE / PubMed, PsycINFO, Cochrane CENTRAL, Google Scholar, Embase y Web of Science utilizando términos basados en palabras clave y términos de encabezamientos de materias médicas (MeSH) antes de adaptarlos a cada base de datos. Se extrajeron datos para los factores clave del estudio (p. ej., población del estudio, ubicación geográfica, diseño del estudio) y los hallazgos principales. Esta búsqueda produjo once artículos: diez manuscritos y un resumen de conferencia. De ellos, dos eran estudios controlados aleatorizados, dos eran intervenciones, tres eran estudios cualitativos y cuatro eran estudios transversales. Los estudios estaban repartidos por todo el país, pero la mayoría se realizaban en las principales áreas metropolitanas. De los artículos incluidos en esta revisión, los hallazgos fueron inconsistentes en la asociación entre el apoyo social; algunos estudios mostraron hallazgos nulos, otros que sólo ciertas fuentes de apoyo social eran significativas y otros que existía una asociación significativa entre el apoyo social y el uso de la PrEP. Esta revisión pone de manifiesto la complejidad de la relación entre el apoyo social y el uso de la PrEP entre los HSH, lo que indica la necesidad de realizar más investigaciones para identificar los tipos y las fuentes de apoyo específicos que mejoran de forma efectiva la aceptación y el cumplimiento de la PrEP. Las intervenciones específicas basadas en estos conocimientos podrían reducir significativamente la incidencia del VIH en la población.

## Introduction

An estimated 1.2 million individuals are living with human immunodeficiency virus (HIV) in the United States [1]. Sexual minority men (SMM) make up a disproportionately high rate of HIV incidence, accounting for 48% of all new HIV cases in 2019 [2]. In 2012, the landscape of the HIV epidemic changed due to the approval of pre-exposure prophylaxis (PrEP), the antiretroviral combination of emtricitabine and tenofovir disoproxil fumarate (Truvada ^®^; F/TDF), by the US Food and Drug Administration to prevent an individual from contracting HIV [3]. PrEP has since been found to be highly effective in preventing HIV infection among those who take it as prescribed [4–9]. Despite this, PrEP uptake has been slow; over 1.1 million individuals are estimated to have indications for PrEP, but only an estimated 224,000 individuals (around 20%) have a prescription [10, 11].

Although the awareness and usage of PrEP have increased since the time PrEP was approved in the US [12], there remain myriad barriers to accessing and remaining on PrEP for many SMM. Structural-level barriers to PrEP include limited access to healthcare services, a lack of health insurance, the inability to pay for a PrEP prescription, and a lack of providers with PrEP knowledge [13–19]. Moreover, previous studies have shown that culturally insensitive healthcare experiences concerning HIV stigma, racism, and homophobia increase medical mistrust and reduce the likelihood of engaging in PrEP or other preventative HIV behaviors among SMM [20]. Individual-level psychosocial barriers include a lack of knowledge about PrEP and concern for PrEP side effects. Further, there is a societal stigma associated with taking PrEP, wherein PrEP has been associated with promiscuity, infidelity, and homosexuality [21–26].

Fortunately, social support may be a factor that can mitigate some of these barriers to increase PrEP use and adherence among SMM. Social support is defined as any communication from providers to a recipient that decreases uncertainty and increases the recipient’s personal control in a situation [27]. Social support may arise from different people in one’s social network and can express itself in different ways. Social support theory posits that there are three main forms of social support that can impact health behavior: *emotional support* (esteem, concern) *instrumental support* (tangible resources such as money, aid, time), or *informational support* (education) [28]. Social support may also come from formal sources, such as professional health providers (e.g., counselors or therapists) [29, 30]. Among the lesbian, gay, bisexual, transgender, queer, and more (LGBTQ+) community, a “chosen family” or “gay family”, groups comprised of other members of the LGBTQ + community, may provide social support in the face of parental rejection for identifying as SMM [31, 32]. Older members of a chosen family may act as mentors toward younger members, and all members may provide social support to one another [33]. There are also identity-specific subgroups within the LGBTQ + community that may provide additional social support to SMM who identify with that subgroup (e.g., house and ball community, drag, bear, leather, etc.) [33].

Previous studies have shown that increased social support has been associated with increased PrEP use in other populations [34]. The mechanism through which social support increases PrEP use and adherence may be through reducing stigma and increasing mutual learning in a social network [35–37]. However, methodological limitations have constrained the state of knowledge regarding the role of social support in fostering PrEP use among SMM. Further, inconsistent measurement of social support has limited our understanding of the underlying mechanisms or causal factors that serve to increase PrEP use. Varying assessments of social support among SMM have also limited our understanding of the impact that distinct types and sources of support received, as well as the perceived quality of that support, has on PrEP use among SMM. This research is needed to identify modifiable social support intervention targets for increasing PrEP use among SMM.

Given that SMM are disproportionately affected by HIV, it is crucial to fully elucidate factors such as social support that have been shown to strengthen engagement in PrEP use. To date, a scoping review has not been conducted to describe the various pathways and overall impact that social support has on PrEP use behaviors among SMM in the United States. The objective of this scoping review is to summarize the current literature base investigating the association between social support and PrEP use among SMM in the United States. Scoping reviews are appropriate for locating and summarizing literature on emerging topics and identifying gaps, such as how social support is associated with PrEP use [38–40].

## Methods

### Search Strategy

Following Preferred Reporting Items for Systematic Reviews and Meta-Analyses (PRISMA) [41] guidelines, a systematic search was performed for studies investigating the association between constructs of the Social Support Theory and PrEP use and adherence among SMM in the US. The search for peer-reviewed articles was conducted on October 27, 2022, using six different databases: MEDLINE / PubMed, PsycINFO, Cochrane CENTRAL, Google Scholar, Embase, and Web of Science. The search was conducted using terms established by using keywords and medical subject headings (MeSH) terms before being adapted to each database (Table 1). Terms were established for SMM, PrEP, and social support terms. Within each category, the Boolean operator OR was used to expand the search, and then combined using the AND term for the three categories.

**Table 1:** Systematic search terms

Database	Search terms	Results	Duplicates	Total
MEDLINE / PubMed	(“homosexuality, male“[MeSH Major Topic] OR “Sexual and Gender Minorities“[Mesh] OR “men who have sex with men“[All Fields] OR “sexual minority men“[All Fields] OR “gay and bisexual men”) AND (“social support“[All Fields] OR “instrumental support” OR “appraisal support” OR “emotional support” OR “informational support” OR “Social Support“[MAJR] OR “social support“[MeSH Major Topic]) AND (“pre-exposure prophylaxis” OR “prep”)	42	0	42
PsycINFO	prep OR pre-exposure prophylaxis AND social support OR emotional support OR instrumental support OR appraisal support OR informational support OR tangible support AND MA homosexuality, male OR MA (sexual and gender minorities) OR men who have sex with men OR sexual minority men OR gay and bisexual men	27	19	8
Cochrane CENTRAL	MeSH descriptor [Pre-Exposure Prophylaxis] explode all trees AND social support OR emotional support OR instrumental support OR appraisal support OR informational support OR tangible support AND MeSH descriptor: [Homosexuality, Male] explode all trees	9	3	6
Google scholar	“men who have sex with men”, OR “sexual minority men” OR “gay and bisexual men”, AND “pre-exposure prophylaxis”, OR “prep”, AND “social support” OR “emotional support” OR “instrumental support” OR “informational support” OR “appraisal support” OR “tangible support” (‘male homosexuality’/exp OR ‘male homosexuality’ OR ‘sexual and gender minority’/exp OR ‘sexual and gender minority’ OR ‘men who have sex with men’/exp OR ‘men who have sex with men’ OR ‘sexual minority men’ OR ‘gay bisexual men’) AND (‘social support’/exp OR ‘social support’ OR ‘emotional support’/exp OR ‘emotional support’ OR ‘appraisal support’ OR ‘informational support’/exp OR ‘informational support’ OR ‘instrumental support’/exp OR ‘instrumental support’ OR ‘tangible support’) AND (‘pre-exposure prophylaxis’/exp OR ‘pre-exposure prophylaxis’ OR ‘prep’) ((TS=(prep or pre-exposure prophylaxis)) AND TS=(homosexuality or sexual and gender minorities or men who have sex with men or sexual minority men or gay bisexual men)) AND TS=(social support or instrumental support or appraisal support or emotional support or informational support or tangible support)	119	8	111
Embase	“men who have sex with men”, OR “sexual minority men” OR “gay and bisexual men”, AND “pre-exposure prophylaxis”, OR “prep”, AND “social support” OR “emotional support” OR “instrumental support” OR “informational support” OR “appraisal support” OR “tangible support” (‘male homosexuality’/exp OR ‘male homosexuality’ OR ‘sexual and gender minority’/exp OR ‘sexual and gender minority’ OR ‘men who have sex with men’/exp OR ‘men who have sex with men’ OR ‘sexual minority men’ OR ‘gay bisexual men’) AND (‘social support’/exp OR ‘social support’ OR ‘emotional support’/exp OR ‘emotional support’ OR ‘appraisal support’ OR ‘informational support’/exp OR ‘informational support’ OR ‘instrumental support’/exp OR ‘instrumental support’ OR ‘tangible support’) AND (‘pre-exposure prophylaxis’/exp OR ‘pre-exposure prophylaxis’ OR ‘prep’) ((TS=(prep or pre-exposure prophylaxis)) AND TS=(homosexuality or sexual and gender minorities or men who have sex with men or sexual minority men or gay bisexual men)) AND TS=(social support or instrumental support or appraisal support or emotional support or informational support or tangible support)	89	39	50
Web of science	“men who have sex with men”, OR “sexual minority men” OR “gay and bisexual men”, AND “pre-exposure prophylaxis”, OR “prep”, AND “social support” OR “emotional support” OR “instrumental support” OR “informational support” OR “appraisal support” OR “tangible support” (‘male homosexuality’/exp OR ‘male homosexuality’ OR ‘sexual and gender minority’/exp OR ‘sexual and gender minority’ OR ‘men who have sex with men’/exp OR ‘men who have sex with men’ OR ‘sexual minority men’ OR ‘gay bisexual men’) AND (‘social support’/exp OR ‘social support’ OR ‘emotional support’/exp OR ‘emotional support’ OR ‘appraisal support’ OR ‘informational support’/exp OR ‘informational support’ OR ‘instrumental support’/exp OR ‘instrumental support’ OR ‘tangible support’) AND (‘pre-exposure prophylaxis’/exp OR ‘pre-exposure prophylaxis’ OR ‘prep’) ((TS=(prep or pre-exposure prophylaxis)) AND TS=(homosexuality or sexual and gender minorities or men who have sex with men or sexual minority men or gay bisexual men)) AND TS=(social support or instrumental support or appraisal support or emotional support or informational support or tangible support)	133	42	91
**Total**		419	111	308

### Inclusion Criteria and Selection Process

There were two phases of applying the selection criteria. Studies first had to meet the inclusion criteria during the title and abstract screening. The article must (1) not have a definitely irrelevant title, (2) be in English, (3) take place in the United States, (4) include sexual minority, cisgender, adult, HIV-negative men, (5) include PrEP use/adherence terms, and (6) include social support terms. Studies that progressed to the full-text screening criteria phase were included in the final analysis if they met the additional inclusion criteria that the full article must (1) be in English, (2) take place in the United States, (3) include sexual minority, cisgender, adult, HIV-negative men, (4) be a study (e.g., not a commentary, editorial, etc.), (5) measure PrEP use/adherence as an outcome, (6) assess social support as an exposure, and (7) assess the association between social support and PrEP use/adherence. There were no exclusions based on the date of publication. Three researchers (CN, HR, OJ) independently performed each extraction step before meeting and compared what studies they deemed appropriate to include. Any discrepancies were discussed and decided between the three researchers. Approval by the university’s Institutional Review Board (IRB) was not required.

## Results

**Fig. 1:**
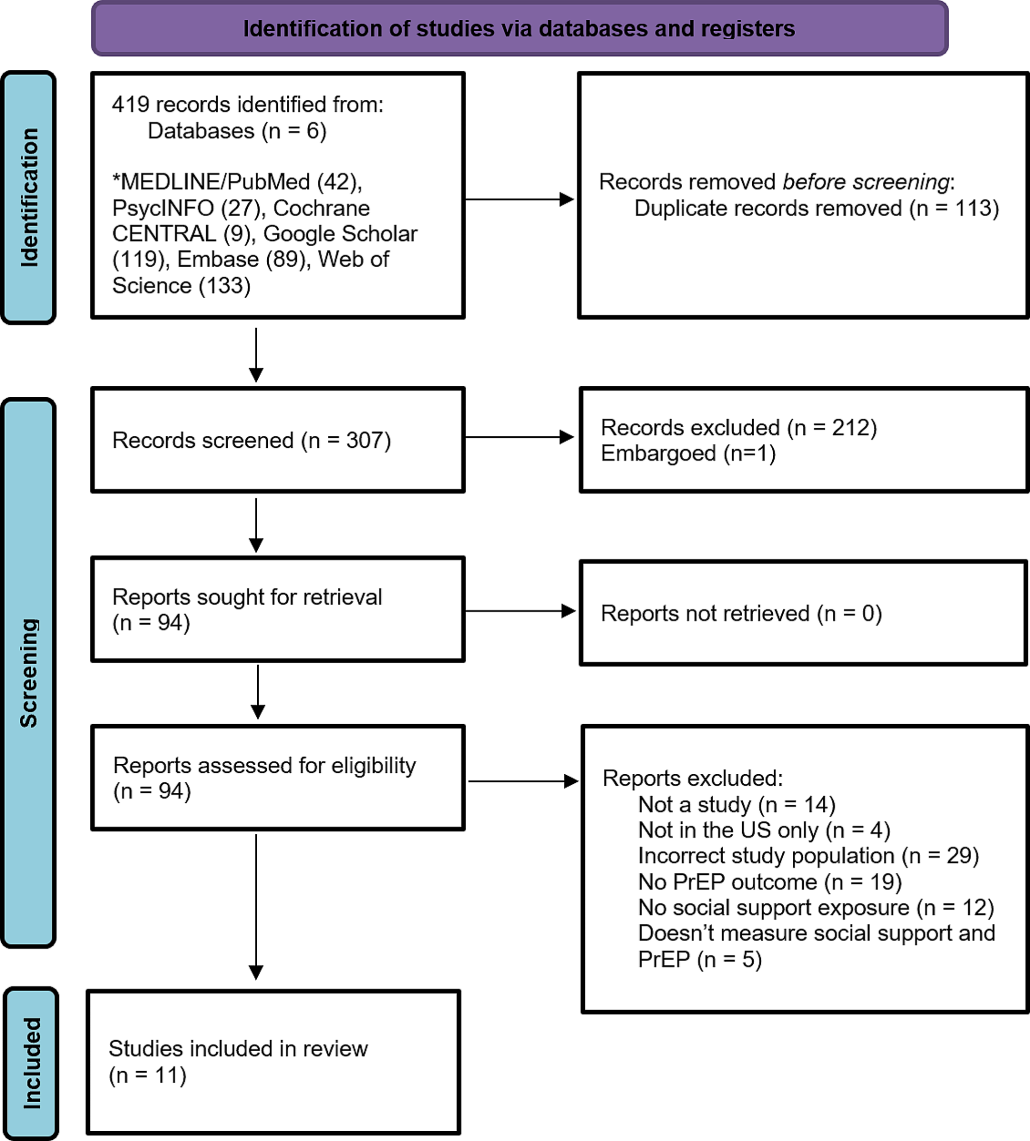
PRISMA diagram [65] for systematic search

Our search yielded 419 articles (Fig. 1). After removing a total of 113 duplicates, 307 articles progressed to the first step of title and abstract screening. This first phase excluded 212 articles based on the title/abstract screening criteria, and one article was excluded as it was embargoed, leaving 94 articles to undergo full-text screening. Of these, 11 studies were included for data extraction in this scoping review [42–52].

### Description of Studies

Eleven articles were included in the data analysis: ten manuscripts and one conference abstract (Table 2). The study designs were diverse, with three qualitative interview studies [48, 50, 52], four cross-sectional studies [42–44, 47], two interventions [46, 49], and two randomized control trials [45, 51]. The studies took place in varied geographic locations across the US. Four took place in the mid-Atlantic region [42, 44, 47, 51]: one in Washington, DC [51] and three from the same study in the metropolitan areas of Philadelphia, PA, Baltimore, MD, and Washington, DC [42, 44, 47]. These three studies used different inclusion/exclusion criteria and thus had different datasets [42, 44, 47]. Three other studies took place in the Midwest [46, 48, 52]: two in Milwaukee, WI [46, 52], and one in both Milwaukee, WI and Cleveland, OH [48]. Additionally, two studies took place in Los Angeles, CA [45, 49], one took place in Jackson, MS [43], and one spanned several cities across different regions of the US, including Providence, RI, Jackson, MS, and St. Louis, MO [50]. Notably, all studies were centered in major metropolitan areas. Six articles included only Black or African-American men in their study [43, 45, 48] or had a high proportion of Black or African-American participants [46, 49, 51]. While the majority of studies included both SMM who were using PrEP and those who were not, two studies included only those who were actively taking PrEP [50, 52], and one intervention included only SMM who were not actively using PrEP [45]. A summary of all article measurements and findings is below (Table 3).

**Table 2:** Study characteristics, population, and methodology

First author, year	Study design	Geographic location	Description of population	Recruitment and sampling method
Bonett, 2022 [42]	Cross-sectional	Metropolitan areas of Philadelphia, PA, Baltimore, MD, and Washington, DC	*N* = 281 cis-gender men that are self-reported HIV-negative or status unaware and had sex with a man in the past 6 months. The median (IQR) age was 23 (20,24) and participants were mostly Non-Hispanic White (64%)	Online recruitment using ads on Grindr and Facebook
Burns, 2021 [43]	Cross-sectional	Jackson, MS	*N* = 142 Black / African American cisgender men, reported sex with another man in the last 12 months, were HIV negative, and no other race (excluding Non-Hispanic Black). Mean age was 27.6 (SD 8.3). Many reported an income below the federal poverty line (60.6%), had up to a high school diploma (55.5%), had health insurance (69%), had used health services in the past 12 months (54.9%), and had not used PrEP in the past 6 months (58.4%). Most did not experience incarceration (93.7%) or housing insecurity in the past 12 months (83.8%)	Convenient and respondent-driven sampling. Recruitment took place at HIV clinics, AIDS service organizations, community-based organizations, social media, and word of mouth
Flores, 2020 [44]	Cross-sectional	Metropolitan areas of Philadelphia, PA, Baltimore, MD, and Washington, DC	*N* = 222 HIV-negative or status unaware cisgender men, reported sexual intercourse with a man in the past 6 months. The mean age was 22.43 (SD 2.07). Around a third identified as a racial / ethnic minority (34.7%), were in a relationship (38.3%), and were currently on PrEP (32.4%). A majority had a college education or higher (60.4%), private health insurance (88.3%). About half were fully employed (50.5%)	Online recruitment using Grindr and Facebook
Harawa, 2020 [45]	Randomized control trial	Los Angeles County, CA	*N* = 61 Black/African American men that were HIV-negative or status unaware and had sex with a man or trans woman in the last 6 months. Mean age 44.3 +/- 11.2 years. Although 72% had completed high school, only 39% were employed or students. Monthly income was < $1,000 for 74% of participants and 31% considered themselves homeless. Most had experienced incarceration (70%).	Direct outreach at public venues, community orgs, parks, and events; provider referrals and fliers; limited online recruitment (Craigslist, Instagram, study website).
Kelly, 2020 [46]	Intervention	Milwaukee, WI	*N* = 37 individuals from 5 social networks. Participants were assigned male at birth, Black/African American or multiracial, 18+, had sex with males in the past year, and were HIV-negative. Participants were on average 27 years old, 89% Black or African American, 73% had high school education or less, and 38% were unemployed.	Recruited 5 seeds in a community venue frequented by Black MSM (e.g., clubs, hangout places, drop-in centers for racial minority LGBTQ + youth). Seeds then invited friends of their network (4–12 members). Members were same except not restricted to race or serostatus.
Meanley, 2021 [47]	Cross-sectional	Metropolitan areas of Philadelphia, PA, Baltimore, MD, and Washington, DC	*N* = 285 cisgender males, 18–25 years old, self-reported as HIV-negative or HIV status unaware. Mean age was 22.18 (SD 2.15), participants were predominantly non-Hispanic white (64.9%), single (64.9%), and had engaged in condomless anal intercourse in the past 3 months (73.3%).	Online recruitment from Grindr and Facebook.
Quinn, 2020 [48]	Qualitative interviews	Milwaukee, WI, and Cleveland, OH	*N* = 46 interviews. Participants were 19–37 Black/African American, gay/bisexual/had sex with another man. Some were current (19.6%) or former (4.3%) PrEP users.	Participants were recruited using purposive sampling through LGBTQ + and HIV organizations, PrEP clinics, or through Facebook
Reback, 2019 [49]	Intervention	Los Angeles County, California	*N* = 129 MSM. Participants were average age 43.3 (12.4), mostly Black/African American (52.7%). Many had more than a high school education (43.4%), were unemployed (72.7%), or were unstably housed (48.1%).	Street- or venue- based outreach, existing HIV testing and counseling programs, flyer distribution, snowball sampling, and community-based organizations.
Rogers, 2022 [50]	Qualitative interviews	Providence, RI; Jackson, MS; Saint Louis, MO	*N* = 33 cisgender MSM that were English or Spanish speaking, reported condomless anal sex with another male in the past 90 days, self-report taking PrEP, and are active in their healthcare. The median age for total sample is 30 (range 20–60), relatively diverse racial/ethnic sample with 54.5% white, 30.3% Black, 15.2% other. Most had private health insurance (75.8%), more than a high school degree (93.9%), employed full time (69.7%).	Three different academic medical centers who had recently initiated PrEP. Participants were recruited from a larger racially, ethnically, and geographically diverse cohort.
Ware, 2021 [51]	Randomized control trial	Washington, DC	*N* = 53. MSM aged 18–29. The average age was 22.5 years and 72% of participants were Black. At enrollment, 96% had ever heard of PrEP, 92% reported condomless anal sex in the prior 3months, of which 36% was with a HIV positive or unknown status man.	N/A
Zapata, 2022 [52]	Qualitative interviews	Milwaukee, WI	*N* = 20 men currently on PrEP. Mean age was 33 (SD 10.48) and ranged from 22–70 years old. Most participants were Caucasian (60%), politically liberal or very liberal (65%), and single (55%). Over 70% reported having missed taking their PrEP at least once in the prior month.	Purposeful sampling recruited from a PrEP clinic database housed at the Medical College of Wisconsin.

IQR, interquartile range; SD, standard deviation

**Table 3:** Study measurements and main findings

First author, year	PrEP use measurement	Social support measure	Analytic methods	Main findings
Bonett, 2022 [42]	PrEP continuum: (1) PrEP unaware, (2) PrEP aware but unwilling to use, (3) PrEP aware and willing to use, but no intention to use PrEP, (4) PrEP aware, willing, and intending to seek PrEP within three months, and (5) current PrEP users.	Social identity support variable made form (1) gay community attachment, (2) sexual orientation disclosure, and (3) emotional support	Structural equation modeling, confirmatory factor analysis	There was a non-statistically significant direct result between social identity support and PrEP engagement (0.10 [− 0.12, 0.32]) There was an indirect but statistically significant association between social identity support and PrEP engagement (0.18 [0.09, 0.28]), via PrEP use in network
Burns, 2021 [43]	PrEP use in the last 12 months (yes/no)	Social support was measured using a previously validated four-item Emotional Support Scale, Cronbach α of 0.84. (Krause, 1995).	Univariate and bivariate analyses using SAS; multilevel logistic regressions	At the environmental level, there was a non-statistically significant result between social support cluster mean and PrEP use 0.78 (0.6, 1.01) At the individual level, there was a non-statistically significant results between social support and PrEP use 0.93 (0.81, 1.07)
Flores, 2020 [44]	Current PrEP use, PrEP use before sexual or drug use	Family Social Support was based on one item that asked participants to indicate the extent to which they received social/emotional support from their family (0 = Not at all, 1 = A little, 2 = Somewhat, 3 = A lot). Other items included comfort with parent-child sex communication, family disclosure of sexual orientation, family prioritization	Descriptive statistics, correlation tests to assess for multicollinearity, logistic regression	Family social support was not significantly associated with current PrEP use. However, comfort with parent sex communication was significantly associated with odds of current PrEP use in the final adjusted multivariable model (aOR = 1.87 (1.18–2.98, *p* = 0.008).
Harawa, 2020 [45]	PrEP awareness and use	Social support was an intervention component provided from social / educational group outings, and by a Peer Mentor pairing who provided support, encouragement, and navigation.	Descriptive statistics and either generalized linear mixed models with a logit link function or logistic regression, depending on the outcome variable	The Peer Mentor intervention arm had PrEP awareness and use increase from 0–22% compared to the control arm whose use increased from 0–9%. In logistic regression, the odds of current usage of PrEP was 0.39 (0.03–4.85) times as likely among Peer-Supported group compared to non-Peer-Supported (p-value 0.426).
Kelly, 2020 [46]	Currently taking PrEP (yes/no)	Social support was an intervention component provided in weekly group sessions plus two biweekly booster sessions. Sessions focused on inspiring / energizing network leaders to help friends avoid HIV infection by learning about PrEP, role-played conversations, learned about PrEP services, normalizing PrEP, and developing plans to assist friends in taking PrEP.	Multilevel models to assess PrEP over time. Included network as a fixed factor; linear, logistic, or negative binomial regression coefficients. ITT analysis.	From baseline to 3 months follow up, 3 participants initiated PrEP use between baseline and follow-up, increasing the percentage using PrEP from 3% (*n* = 1) to 11% (*n* = 4).
Meanley, 2021 [47]	Lifetime PrEP use ever (yes/no)	Gay community attachment was defined by four variables: “I feel that I am part of my area’s LGBTQ + community,” “Participating in my area’s LGBTQ + community is a positive thing for me,” “I feel a bond with the LGBTQ + community,” and “I am proud of my area’s LGBTQ + community”). Response options were on a 4-point scale (0 = Strongly disagree, 3 = Strongly agree), Cronbach’s α = 0.86.	Bivariate associations (chi-square, t-tests, Pearson’s correlation) and structural equation modeling, adjusting for age, race / ethnicity, sexual orientation, employment status, relationship status, and recent condomless intercourse.	Higher scores on gay community attachment were directly associated with lifetime PrEP use.
Quinn, 2020 [48]	Perceptions of PrEP and PrEP use among peers	Social and cultural factors (e.g., How would you describe the gay or LGBTQ + community in Milwaukee / Cleveland), friends, and peer groups (e.g., Thinking about your friendship circle or peer group, what percentage of them from 0 to 100 are gay / bisexual / transgender).	Team-based multi-stage analytic coding strategy to organize the data, create a codebook, refine the codebook, use axial coding to finalize the codebook, apply the codebook, and finally use thematic content analysis to identify themes.	The key themes were (1) how peers talk about PrEP, (2) filling gaps left by healthcare providers, (3) peers improved trustworthiness of PrEP, (4) reducing stigma and changing the narrative around PrEP, (5) a need for more leaders in the Black GBM community. Participants stated that friends, peers, and constructed or gay families were the primary source of PrEP information, which influenced how they viewed PrEP. Gossip, rumors, and stigma were barriers to PrEP, but openly discussing PrEP had the power to increase comfort and social change around PrEP. This filled knowledge gaps from their healthcare and medical providers. Participants also trusted their friends, peers, and constructed families more than healthcare providers, as the population has a lot of medical / pharmaceutical mistrust. Participants noted the need for identifying “movers and shakers”, leaders, or role models in the community.
Reback, 2019 [49]	Still on PrEP, number of missed doses in the past 4 days, number of missed doses in the past 30 days, and if they had not taken PrEP for 4 + days in a row.	A.S.K.-PrEP is a five-session peer navigator service program that took place over a 3-month period that was adapted from ARTAS (Antiretroviral Treatment Access Study), the CDC intervention for linkage to HIV care. The sessions consisted of a PrEP Knowledge Pre-test, assessing participant readiness for PrEP adherence, planning for adherence and removing any barriers to taking PrEP, and continue PrEP adherence. They also received adherence support text messages based on Social Support Theory, Health Belief Model, or Social Cognitive Theory.	Descriptive analysis	91.5% of MSM were successfully linked to PrEP care in a median number of 9 (IQR 4–15) days. 69.6% reported that they were still taking PrEP. 83.3% of MSM reported that they did not miss one PrEP dose in the previous 4 days, and 48.7% reported that they did not miss one PrEP dose in the previous 30 days. About 20% of participants (MSM: 19.2%) reported that they did not take a PrEP dose on four or more consecutive days in the previous 30 days. 46.5% of MSM elected to receive the adherence support text messages; these MSM were more likely to report that they still took PrEP medication than participants who did not receive the text messages (85.7% vs. 61.4%; χ^2^(1) = 12.0, *p* < 0.001). The association between text message support and PrEP adherence was significant for MSM (81.1% vs. 59.3%; FET, *p* = 0.014)
Rogers, 2022 [50]	Factors related to PrEP, costs associated with PrEP, pharmacy and mail order experiences, impact of COVID-19 on PrEP, thoughts on “next generation” PrEP, beliefs, and attitudes about PrEP as an HIV prevention / health promotion tool	Factors related to PrEP included social factors such as discrimination, their own experience with PrEP, and others’ experiences with PrEP	Deductively created a codebook, inductively coded emergent codes, themes, patterns, and conclusions	Participants reported that being active and supported in the LGBTQ + community normalized PrEP and therefore reduced the stigma associated with taking PrEP and provided support to engage and adhere to a PrEP regimen. They also felt that any person who was supportive in friendship or romantic relationships was an important factor for participants to stay on PrEP. Participants also reported that having access to medical providers who affirmed their sexual orientations and were knowledgeable about PrEP made them feel comfortable to ask questions about PrEP and improve their healthcare experience.
Ware, 2021 [51]	Adherence measured using DBS TFVdp	One of the three arms of this RTC was a bidirectional Facebook group	Descriptive analysis	Protective PrEP adherence DBS TFVdp levels were measured in 46% of the Financial Incentive group, 57% of the Social Media group, and 67% of the Control group (*p* = 0.38). Both the financial and social media arms had no statistical impact on PrEP adherence compared to the control arm.
Zapata, 2022 [52]	Time on PrEP	Interviews inquired about level of social support, social support involvement in care, social and PrEP-related stigma, and sharing their PrEP use with people in their life.	Guided by Grounded Theory, researchers took notes on all interviews, grouped concepts into categories, coded interviews using the category list, then refined thematic categories using comparative analysis.	Main themes included PrEP related stigma, relationship-related stigma, social support within the LGBTQ + community, psychological impact of PrEP, and access to care because of stigma. PrEP related stigma: Participants said that people in their networks often see PrEP as a gateway to have unprotected anal sex and have multiple partners. Some said this led them to hide their PrEP use. Some said this led them to attempt to increase empowerment around PrEP users. Relationship-related stigma: Participants felt that revealing they were on PrEP led others to judge their non-monogamous relationship status or felt conflict in a relationship for choosing to stay on PrEP. Social support within the LGBTQ + community: Many participants learned about PrEP from other queer men, leading them to seek out care from LGBTQ+-friendly medical providers so they would not feel judged or lectured. Establishing a well-known care network was critical. However, some participants reported encountering medical providers who did not know about PrEP and identified that as a significant barrier. Lack of communication with a provider in general felt judgmental or discriminatory. Discussing PrEP with other LGBTQ + men normalized PrEP use and made participants feel like a resource to their community, and provided and received companionship, informational support, and emotional support from their peers.

aOR, adjusted odds ratio; GBM, gay and bisexual men; CDC, Centers for Disease Control and Prevention; MSM, men who have sex with men; FET, Fisher’s exact test; DBS TFVdp, dried blood spot Tenofovir diphosphate

### Qualitative Findings

Three qualitative interview studies in this review produced similar overarching thematic categories encompassing social factors that influence PrEP use. These include the perceived stigma that PrEP use equates to promiscuity, supportive and unsupportive healthcare providers, support from the LGBTQ + community or chosen families, and support from romantic relationships. These underscored the importance of both social support and social networks and their potential positive influence on PrEP knowledge, uptake, and adherence [48, 50, 52]. Specifically, discussions of PrEP within a social network positively contributed to the normalization of PrEP, thereby increasing self-efficacy to initiate PrEP use [48, 50]. Participants further highlighted that being an active member of their LGBTQ + community led to a reduction in PrEP-related stigma. Overall, discussing PrEP with other LGBTQ + men provided companionship, educational/informational support, and validation and therefore normalized PrEP use, leading participants to perceive the medication as a resource [48, 50, 52].

Inversely, participants in two studies who belonged to multiple marginalized groups (e.g., racial/ethnic minority and sexual minority) noted that discussing PrEP use within their social networks increased the level of stigma experienced around the use of PrEP and its perceived association with promiscuity, contributing to gossip and rumors that increased experiences of stigma and served as barriers to PrEP use [48, 52]. Some partnered participants also reported that initiating PrEP in the context of monogamous relationships may be seen as a sign of being unfaithful or unethically non-monogamous, both of which have the potential to elicit anger or judgment from their partner or the wider community [52]. A reported solution to this may be an increase in partnered peer mentors with experience taking PrEP [48].

Notably, a universally present theme for PrEP interventions focused on engaging a sexually affirming social network of healthcare providers [48, 50, 52]. Some participants were also of the belief that, if healthcare providers and personnel were better equipped to educate patients on the benefits and process of taking PrEP, the community would become less reliant on their social networks for the same information [52].

### Observational Findings

Among the cross-sectional studies, there were inconsistent findings regarding the relationship between social support and PrEP use. Most of the studies found null or non-statistically significant results. One study investigated the association between social identity support and PrEP engagement among young SMM in Philadelphia, Baltimore, and Washington, DC through an online survey. The investigators measured social identity support by creating a latent variable from three constructs: gay community attachment, sexual orientation disclosure, and emotional support. Using structural equation modeling to assess the relationships between social identity support, PrEP norms, and economic instability, the investigators did not find a statistically significant direct association between social support and PrEP norms (β = 0.10, 95% CI: -0.12–0.32). However, there was a significant indirect association between social identity support and PrEP engagement mediated by descriptive PrEP norms (β = 0.18, 95% CI: 0.09–0.28) [42].

In another study, investigators assessed whether participants’ family social support and comfort with parent-sex communication were associated with PrEP uptake among young SMM. Family social support was from one question asking participants the extent to which they received social/emotional support from their family (0 not at all, 1 a little, 2 somewhat, 3 a lot). To measure comfort with parent-sex communication, participants rated the extent to which they felt comfortable about their sexual behaviors, sexual identity/attraction, sexual health, and taking PrEP with each of their parents respectively (0 not at all, 1 a little, 2 somewhat, 3 very). Although family social support was not significantly associated with current PrEP use, after adjusting for family dynamics and demographics, there was a significant association between comfort levels with communication about sex with parents and current PrEP use (aOR 1.87; 95% CI: 1.18–2.98; *p* = 0.008) [44].

A third analysis conducted in the same mid-Atlantic corridor among young men who have sex with men (YMSM) used structural equation modeling to test pathways between PrEP stereotypes, stigma, lifetime PrEP use, and gay community attachment. Gay community attachment was comprised of four questions about their area’s LGBTQ + community (e.g., “Participant in my area’s LGBTQ + community is a positive thing for me”). Higher gay community attachment was significantly and directly associated with lifetime PrEP use (β = 0.25, 95% CI: 0.11–0.40, *p* = 0.001). Gay community attachment was a measure developed by the authors comprised of variables related to feeling like part of the area’s LGBTQ + community, feeling like participating in said community was a positive experience, feeling a bond with the community, and feeling proud of the community [47].

Finally, the fourth and final cross-sectional study investigated factors associated with PrEP use among Black / African American SMM in Jackson, MS, including social support. This analysis was conducted at the individual level and the zip code level. Social support was measured using the four-item Emotional Support Scale [53]. After adjusting for covariates, the authors found that there were no statistically significant associations between social support and PrEP use at the individual level, (aOR 0.93, 95% CI: 0.81–1.07, *p* = 0.31). Similarly, there was no significant association between social support measured in a cluster mean at the zip code level (aOR 0.78, 95% CI 0.6–1.01, *p* = 0.06) [43].

### Experimental Findings

The first intervention study investigated a social network intervention that enrolled five participants (i.e., seeds) who were Black, HIV-negative, men who had sex with men [46]. The seeds, in turn, recruited their network members (average: 8, range: 4–12). The social network intervention included five weekly group sessions and two biweekly booster sessions focused on inspiring or energizing network leaders to help their friends avoid HIV infection by learning about PrEP, practicing conversations about PrEP, normalizing PrEP, and developing plans to help their friends adopt PrEP. After the intervention was completed at three months, three of the 27 individuals in the intervention initiated PrEP use, increasing the prevalence of PrEP use from one (3%) to four total participants (12%) [49].

The second intervention study was a peer navigation study [49]. It was adapted from the CDC’s Antiretroviral Treatment Access Study (ARTAS) [54], which successfully linked HIV-positive participants to HIV care. The five-session intervention provided PrEP knowledge, assessing the participant for PrEP adherence, removing any barriers to adherence, and text messages aimed to increase adherence using Social Support Theory, Health Belief Model, and Social Cognitive Theory. 91.5% of SMM were successfully linked to PrEP care in a median number of nine (IQR 4–15) days. Of all participants, 69.6% reported that they were still taking PrEP. 83.3% of SMM reported that they did not miss one PrEP dose in the previous four days, and 48.7% reported that they did not miss one PrEP dose in the previous 30 days. About 20% of participants (MSM: 19.2%) reported that they did not take a PrEP dose on four or more consecutive days in the previous 30 days. Nearly half (46.5%) of MSM elected to receive the adherence support text messages; these MSM were more likely to report that they still took PrEP medication than participants who did not receive the text messages (85.7% vs. 61.4%; χ^2^(1) = 12.0, *p* < 0.001). The association between text message support and PrEP adherence was significant for MSM (81.1% vs. 59.3%; FET, *p* = 0.014) [49].

Finally, two randomized controlled trials met the inclusion criteria in this review. The first compared financial incentives against a social media support group and a control group among young SMM of color in Washington, DC [51]. Participants were randomized 1:1:1 to standard-of-care PrEP (control group), standard-of-care PrEP plus an invitation to a bidirectional Facebook support group supervised by two clinicians (social media group), or standard-of-care PrEP plus a $50 gift card at each of two follow-up visits (financial incentive group). Participant adherence to PrEP was measured using dried blood spot (DBS) tenofovir diphosphate levels. After six months, protective PrEP adherence (≥ 4 doses/week) was measured in 46% of the financial incentive group and 57% of the social media support group compared to 67% of the control group (*p* = 0.38). Both the financial and social media arms had no statistical impact on PrEP adherence compared to the control arm [51].

The second randomized controlled trial assessed the Passport to Wellness intervention, which was comprised of a customized wellness plan, financial incentives, social/educational group outings, and a peer mentor pairing who provided support, encouragement, and navigation [45]. Participants were randomized 1:1 into the Passport to Wellness intervention or to a version that did not include the peer mentor aspect. The peer mentor intervention arm had PrEP awareness and use increase from 0 to 22% compared to the control arm whose use increased from 0 to 9%. However, the results of the generalized linear mixed model were not statistically significant at α = 0.05; the odds of current usage of PrEP was 1.48 (95% CI: 0.31–7.00) as likely among peer-supported group compared to non-peer-supported group (*p* = 0.625) [45].

## Discussion

The systematic search conducted in this scoping review resulted in 11 studies, demonstrating the scarcity of research on the association between social support and PrEP use among SMM. All studies were conducted in major metropolitan areas, with few taking place in the US Southeast region where rates of HIV are among the highest [2]. The findings were mixed on the association between social support and PrEP use and varied from null to positive associations; several studies had null findings [43, 45, 51], some showed that social support, in general, was directly [46, 48–50, 52] or indirectly [42] associated with increased PrEP use or adherence, and others showed that social support from specific members was associated with increased PrEP use [44, 47]. This inconsistency of findings suggests the need for more research on how the different forms and sources of social support may impact PrEP use among SMM.

The qualitative studies in this analysis suggested the substantial importance of social support for use, normalization, and destigmatization of PrEP. This aligns with previous studies that have shown that resistance to HIV-related care is rooted in HIV stigma and a fear of negative consequences from peers [55]. Normalizing HIV-treatment and -prevention services may help an individual feel more comfortable seeking or discussing HIV-related medications such as PrEP. Empowering those who are taking PrEP to disclose their use of PrEP, when safe to do so, and share their experiences with other members of their social networks could be one way of reducing stigma and increasing the acceptability of PrEP [34, 48, 56]. Previous studies have shown normalizing HIV care may act as a protective factor against the internalization of sex-negative attitudes and improve an individual’s health outcomes and self-care practices [57, 58].

The *source* providing social support to SMM may also make a difference; two studies described the importance of the gay community [47] and parent-child social support [44]. Receiving social support from another individual in one’s gay community may help to normalize PrEP if that individual has favorable views of PrEP or is taking PrEP themselves, which has been associated with higher PrEP adherence [9, 59]. A key strategy to normalize PrEP is to also do so in clinical settings through LGBTQ-affirming healthcare providers and using standardized approaches to PrEP provision and education [60]. Leveraging a peer-driven approach may be another strategy, as previous studies have shown that having a mentor or role model within one’s identifying community has been beneficial to PrEP use and adherence [61]. In general, further investigation into the specific sources of social support, whether it be from the gay community, parents, or healthcare providers, is warranted.

The experimental studies in this analysis provided social support in a variety of ways: a customized wellness plan intervention informed by Social Support Theory, a social network intervention, a peer navigator intervention, and a bidirectional Facebook group. While the experimental studies did not find any statistically significant associations, one important result that stood out was that individuals who received theory-based text message support were more likely to report taking PrEP medication than participants who did not receive the text messages [49]. Behavioral change interventions benefit greatly from theory, as theoretically informed interventions often lead to better outcomes than those that do not consult theory [62]. Further, this suggests that, given that the social support in this study was provided through text, an anonymous source of social support may be an important strategy to investigate to avoid stigma or judgment from individuals in someone’s social network.

The observational studies in this analysis suggest that the association between social support and PrEP use needs further investigation. While some studies showed a direct association [46, 48–50, 52], Bonett et al. showed an indirect association between social identity support and PrEP engagement through PrEP norms [42]. This suggests that social network members who show support for sexual minorities are likely to influence overall peer norms around PrEP and that positive attitudes about PrEP in networks impact SMM’s decisions to take PrEP. In some models, social support has been shown to serve as a moderator; the stress-buffering theory posits that supportive social interactions may provide resources promoting adaptive behavioral responses when faced with stress [63]. One study that aligns with this hypothesis found that more friend-based social support acted as a buffer to above-average minority-identity stress and resulted in little negative effect [64]. The mechanisms behind the relationship between social support and PrEP use warrant further study. Future interventions may benefit from taking a social network approach to further investigate the function and structure of specific individuals on PrEP use and adherence among SMM. Further, these results indicate that quantitative measures may not be adequately capturing the nuanced ways that social support may bolster PrEP use for some SMM. Studies in this review measure *social support*, which may not necessarily equate to *social support for PrEP use*, given the associated stigma. The development of a PrEP-specific support measurement may be warranted.

These studies explore whether support from one’s social network members is associated with active participation in the HIV-prevention continuum through PrEP use and adherence, a critical effort to strengthen prevention efforts among SMM. These findings suggest a need to further investigate the specific ways that supportive members of social networks can influence SMM towards or away from using PrEP. Members of networks may be viewed as highly supportive in some ways (e.g., instrumental support), but less supportive or even stigmatizing about sexuality and/or the use of PrEP. Further study is warranted to identify the best forms and sources of PrEP-specific support to inform future interventions. For example, future interventions may benefit from social support aimed at reducing the stigma associated with PrEP and HIV. Social support and related factors work together to impact PrEP use. Once the mechanism of social support is better understood, researchers can develop meaningful interpersonal / community-based interventions that leverage those mechanisms of social support that help increase PrEP use and adherence in efforts to increase HIV prevention.

### Limitations

There were several limitations to this review. The scope of this review was limited to the US and cisgender sexual minority men, limiting its application to other populations. Given the high variance in social determinants of health for different geographic areas and transgender populations, different reviews should be done for these populations, as their experiences are very different from this population. The studies included in this review were all conducted in major metropolitan areas and primarily in the northern half of the country, which is a major gap. However, many studies did prioritize Black and African American participants, which is a disproportionately affected group that should be emphasized. Further, only one of the studies in this analysis measured PrEP using dried blood spot scores, while the rest were self-reported measures. More studies are needed where PrEP use and adherence are confirmed via biomarkers.

## Conclusion

The findings of this review highlight the complexity of the relationship between social support and PrEP use among SMM. While some studies have demonstrated a significant positive impact of social support on PrEP uptake and adherence, others showed null results. This inconsistency underscores the need for further research to elucidate the specific types and sources of PrEP-specific social support that most effectively promote PrEP use. Understanding these nuances can inform targeted interventions aimed at increasing PrEP utilization among SMM, ultimately contributing to the reduction of HIV incidence in this population.

Mixed results suggest social support may be highly nuanced and subjective for SMM and that a one-size-fits-all approach is likely not an effective way for social support or social network-based interventions to increase PrEP use. Further study is needed to assess the forms, functions, and quality ratings of social support for PrEP use among SMM. Additional study of the pathways and mechanisms by which social support bolsters or hinders PrEP use is also needed. Some members of SMM’s social networks may have less favorable views or stigmatizing attitudes towards PrEP, which may influence SMM’s comfort on discussing PrEP with their network members. Inversely, some studies demonstrated that SMM may have network members that are taking PrEP or have favorable views toward PrEP, which may increase the likelihood of discussing the medication. Previous studies have shown that having even one social network member increases one’s willingness to take PrEP themselves [56]. Social support-based interventions that seek to reduce PrEP stigma and increase social norms around PrEP use may be particularly important to investigate. Further, couples-based interventions that seek to normalize and reframe narratives around the use of PrEP as one potential method of loving and protecting one another in the context of supportive relationships are another potential avenue to pursue.

The current review suggests there may be paradoxical findings around how social network members can both help and hinder PrEP use and that these nuances need to be studied further. Moreover, social support and social network-based interventions focused on increasing PrEP uptake among SMM are likely to be most efficacious if interventionists can help participants identify the specific form of social support that would be helpful, offered by whom deemed most appropriate, and under what circumstances.
